# Altered Functional Network Affects Amyloid and Structural Covariance in Alzheimer's Disease

**DOI:** 10.1155/2018/8565620

**Published:** 2018-12-02

**Authors:** Ya-Ting Chang, Chi-Wei Huang, Wen-Neng Chang, Jun-Jun Lee, Chiung-Chih Chang

**Affiliations:** Department of Neurology, Kaohsiung Chang Gung Memorial Hospital, Chang Gung University College of Medicine, Kaohsiung 83301, Taiwan

## Abstract

**Background:**

We aimed to investigate how altered intrinsic connectivity networks (ICNs) affect pathologic changes of Alzheimer's disease (AD) at a network-based level.

**Methods:**

Thirty normal controls (NCs), 23 patients with AD-mild cognitive impairment (MCI), and 20 patients with AD-dementia were enrolled. We compared the organization of grey matter structural covariance and functional connectivity in ICNs between NCs and all AD patients who were amyloid *β* (A*β*)-positive. We further used seed-based interregional covariance analysis to compare structural and A*β* plaque covariance in default mode network (DMN) between AD-MCI and AD-dementia groups.

**Results:**

The patients with AD had increased functional interregional covariance among the regions of the ICN anchored to dorsal caudate (DC) seeds compared to the NCs. The increased connectivity was associated with extended patterns of reduced A*β* plaque covariance in the AD-dementia group compared to the AD-MCI group within the striatal network anchored to DC seeds. Patterns of lower A*β* plaque covariance in the AD-dementia group compared to the AD-MCI group were more extended within the network anchored to DC seeds than within the DMN, which was undergoing functional failure in the patients with AD. Significant decreased structural covariance in the AD-dementia group compared to the AD-MCI group was more extended in the DMN during functional failure.

**Conclusions:**

Functional connectivity in ICNs affects the topographic spread of molecular pathologies. The temporal trajectory of pathologic alterations can be well demonstrated by pathologic covariance comparisons between different clinical stages. Pathologic covariance can provide critical support to pathologic interactions at network and molecular levels.

## 1. Introduction

The tau propagation theory [1] states that neurofibrillary tangles in Alzheimer's disease (AD) are deposited along intrinsic connectivity networks (ICNs), and interconnected regions with high basal metabolism have been reported to be targets for accelerated pathologic accumulation [2, 3]. Meanwhile, alterations in ICN dynamics are also believed to change the expanding nature of amyloid *β* (A*β*) in AD [3, 4].

Healthy individuals have high functional connectivity in the default mode network (DMN) [5]; nodes within this functional network (FN) have exhibited tightly correlated grey matter volumes [4]. This structural covariance map is referred to as a structural network (SN) [4]. Significantly reduced structural covariance in DMN hubs has been observed in the early stage of AD [6]; however reports on the relationships with functional alternations as revealed by resting state function magnetic resonance imaging (rs-fMRI) have been consistent [6].

*In vivo* amyloid positron emission tomography (PET) has also shown that fibrillar A*β* loads of regions typically involved in the DMN demonstrate high collinearity, the so-called amyloid network (AN) [3]. As the DMN remains a neuroimaging hallmark in the early clinical stage of AD [7, 8], the assessment of functional connectivity and pathologic burden can only be performed at this particular clinical stage [3]. In other words, comparisons of alterations in the SN and AN between different stages of the disease may help to incorporate the concept of ICNs into the spatiotemporal framework of an AD biomarker model [9].

On functional failure in the DMN, ICNs involved with the striatum [10] have been reported to subsequently exhibit increased functional connectivity [11]. Among the striatal networks, the dorsal caudate (DC) and its functional interconnected regions have been shown to be an ideal model to assess cognitive modulation in AD because of the involvement with heteromodal association cortices [12]. Exploring relationships between FN and the molecular pathologic networks anchored to DC seeds [12] may help to elucidate the mechanisms by which alterations in ICNs influence the processes facilitating the pathologic spread in AD.

Regarding the pathophysiologic model of AD, molecular pathology deposits along the ICNs [13], patients with AD have decreased structural covariance among regions of the DMN [6], and progressive network changes in AD [14] are characterized by functional failure in the DMN and a compensatory increased connectivity in other FNs [10, 15]. However, no cross-sectional studies have simultaneously measured AD-associated changes in ANs, SNs, and FNs to directly compare alterations in pathology at a network level.

Seed regions have commonly been used to investigate syndrome-related FNs [4] and SNs [4, 6]. As ICNs have been shown to have a clinical impact [4, 16], the aim of this study was to investigate the relationship between seed volume and cognitive performance. Using rs-fMRI and high resolution structural images in florbetapir (18F-AV-45)-positive AD [17], we explored whether the pattern of decreased structural covariance in patients with AD as compared to normal controls (NCs) was different between the ICNs undergoing functional failure and those undergoing a compensatory increase. We then compared the pattern of A*β* plaque covariance in patients with AD-dementia and AD-mild cognitive impairment (MCI). We hypothesized that high functional connectivity in each ICN would accelerate the accumulation of fibrillar A*β* along the same network through the dementia phase of the disease. Therefore, we investigated whether the pattern of decreased A*β* plaque covariance in patients with AD-dementia, relative to those with AD-MCI, was more extensive within ICNs undergoing compensatory increase than within those undergoing functional failure.

## 2. Methods

### 2.1. Inclusion and Exclusion Criteria

Forty-three patients with AD-MCI or AD-dementia [17, 18] were enrolled from Department of General Neurology of Chang Gung Memorial Hospital from 2011 to 2017. The patients were included based on the consensus of panels comprising neurologists, neuropsychologists, neuroradiologists, and experts in nuclear medicine [19]. A Clinical diagnosis of AD-dementia or AD-MCI was based on the International Working Group criteria with positive PET amyloid imaging of 18F-AV-45 by visual rating [17, 18]. Only the patients with a clinical dementia rating (CDR) scores of 1 were included in the AD-dementia group. Patients were included in the AD-MCI group if they had (1) concerns regarding a change in cognition from the patient or an informant, (2) scores on cognitive tests of approximately 1 to 1.5 standard deviations below the education- and age-adjusted NCs in one or more cognitive domains, and (3) preservation of independence in functional abilities without dementia. The exclusion criteria were a clinical history of stroke, modified Hachinski ischemic score> 4 [20], and depression.

A volunteer group of education- and age- matched NCs, who had no underlying neurological or psychiatric disorders, was recruited from outpatient neurological clinics. The Human Ethics Committee of Chang Gung Memorial Hospital approved the study protocol. All of the subjects or their authorized caregivers provided written informed consent.

### 2.2. Study Design

18F-AV-45 PET, cognitive testing, and magnetic resonance imaging (MRI) were performed within a 4-week period.

### 2.3. Image Acquisition

MR studies were conducted at 3T using a GE Signa Excite scanner (GE Medical System, Milwaukee, WI). The scanning protocol for 3-dimensional (3D) T1-weighted images was as follows: inversion-recovery-prepared, 3D, spoiled, gradient-recalled acquisition in a steady-state sequence with repetition time/inversion time of 8,600 ms/450 ms, 240 × 240 mm field of view, and 1 mm slice thickness. Rs-fMRI scans were performed with the patients' eyes closed using a T2^*∗*^-weighted echo-planar imaging sequence (TR 2500ms, TE 45 ms, FOV 240 × 240 mm, flip angle 10°, thickness 4 mm, and 200 scans of 32 contiguous axial slices) with a total scanning time of 10 minutes per subject.

18F-AV-45 was synthesized at the cyclotron facility of Chang Gung Memorial Hospital. The PET acquisition protocol, optimal scanning time, and image reconstruction were according to a previous study [21].

### 2.4. Data Preprocessing

#### 2.4.1. Structural Volume and PET

3D T_1_-weighted MRI and 18F-AV-45 PET data were preprocessed using Statistical Parametric Mapping 12 (SPM12) software (http://www.fil.ion.ucl.ac.uk/spm/) running under MATLAB 7.9 (MathWorks, Natick, MA).

Preprocessing included realignment, segmentation, normalization into standard stereotactic Montreal Neurological Institute (MNI) spaces, and spatial smoothing using a Gaussian Kernel of 5 mm full-width at half-maximum (FWHM) for T1 and 7 mm FWHM for PET. Using diffeomorphic anatomical registration via the exponentiated lie algebra approach, related tissue segments were used to create a custom template for both T1 and PET. Using REST version 1.8 [22], the PET images were normalized to white matter regions [23]. 18F-AV-45 standardized uptake value ratio (SUVr) of the seed regions was extracted and used in further analysis. The raw volume of interest (VOI) of the hippocampus and total intracranial volume (TIV) were estimated using surface-based atlas maps in Computational Anatomy Toolbox 12 in SPM12 [24]. Hippocampal VOI analysis used the TIV-adjusted volume.

#### 2.4.2. Rs-fMRI

The first 10 volumes of rs-fMRI were discarded to reduce fluctuations in MRI signals. The preprocessing steps of rs-fMRI included slice time correction, realignment, segmentation, normalization into standard stereotactic MNI spaces, and spatial smoothing using a Gaussian Kernel of 6 mm and resampling to 2 × 2 × 2 mm^3^. Subsequently, functional images were smoothed with 6 mm FWHM.

For motion artifacts, the following cut-off scores were used: (1) less than 1% variation in the global average blood oxygen level dependent (BOLD) signal from scan to scan and (2) 0.25 mm/TR of frame-wise displacement. Simultaneously, images were detrended and filtered to 0.008 ~0.09 Hz. After regressing out the head movement time series, white matter and cerebrospinal fluid signals were extracted from each voxel using an anatomical component-based noise correction method as implemented in CONN functional connectivity toolbox (http://www.nitrc.org/projects/conn) [25, 26]. Seed based individual correlation maps using the CONN toolbox were constructed by extracting the mean resting-state BOLD time course from entorhinal seeds and DC seeds. The significance maps of seed-to-voxel connectivity in all AD patients as compared to NCs were computed using second-level analysis of relative functional connectivity using a two-sided independent* t*-test (peak-level at uncorrected* p*< 0.001; cluster-level at false discovery rate [FDR] corrected* p*< 0.05). The correlation coefficients between the seed regions and all the significant clusters represented the voxel-wised functional connectivity for entorhinal seeds and DC seeds. All the resulting coefficients were converted to normally distributed scores using Fisher's transformation.

### 2.5. Neurobehavioral Assessments

We used the Mini-Mental Stage Examination (MMSE) [27] to assess general cognitive function, and functional severity was determined using CDR scores. Episodic memory was assessed using the Chinese version of the verbal learning test (CVVLT) [28], and we recorded the four verbal learning trials of a 9-word list with free recall after 10 minutes (CVVLT-10 min) and cued recall (CVVLT-cued) (number of words recalled with cued procedures over four learning trials). The CVVLT-10 min was used to evaluate delayed recall, and CVVLT-cued was used to measure memory under a cued response. Visuospatial function was assessed by visual object and space perception (VOSP) [29] and modified Rey–Osterrieth (mR-O) complex figure copying [30], and executive functions were assessed using Stroop interference [31] and the Trails Making Test parts B (TMT parts B) [32].

### 2.6. Seed-Based Analyses

Seed-based analyses were performed using two main steps. First, volume and normalized 18F-AV-45 SUVr were extracted from a 4 mm radius sphere around the bilateral entorhinal (±25, -9, -28) [6] and bilateral DC (±13, 15, 9) [33] seeds. Second, statistical contrasts were set to identify the voxels reflecting differences between groups in regression slopes for each seed. Differences in the slopes were referred to as the differences in structural and normalized 18F-AV-45 SUVr covariance [6]. Using the threshold of family-wise error corrected* p*< 0.05 at the voxel level and uncorrected* p*< 0.01 at the cluster level with a cluster size >100 voxels, we established the map of voxels that expressed a stronger structural covariance in the NCs compared to all patients with AD. Using the same threshold, we further established the map of voxels that expressed a stronger structural covariance and A*β* plaque covariance in the patients with AD-MCI compared to those with AD-dementia.

### 2.7. Statistical Analysis

Clinical data were expressed as mean±standard deviation. Student's* t*-test was used to compare continuous variables between the AD-MCI, AD-dementia, and NCs groups, with post hoc Bonferroni correction. Statistical significance was set at* p*<0.05. All statistical analyses were conducted using SPSS software (SPSS version 22 for Windows®, SPSS Inc., Chicago, IL).

## 3. Results

### 3.1. Comparisons of Clinical and Pathologic Data

The demographic data are shown in Table 1. Patients with AD-MCI had significantly lower episodic memory scores and hippocampal volume than the NCs (*p*< 0.05). There were no significant differences between the AD-MCI and NCs groups in other cognitive scores (*p*> 0.05). The AD-dementia group had lower episodic memory scores, hippocampal volume, and all the other cognitive scores compared to the AD-MCI and NCs groups (*p*< 0.05).

### 3.2. Importance of Seed Volume and Its Relationship with Memory Performance

There was a significant positive correlation between right entorhinal seed volume and episodic memory scores including delayed recall and memory under a cued response (*p*< 0.05; Table 2); however the right DC seed volume was only correlated with memory under a cued response (*p*< 0.05; Table 2). Regarding other cognitive functions, the right entorhinal seed volume was only associated with Stroop interference scores (*p*= 0.01; Table 2), whereas bilateral DC seeds were correlated with all executive function scores and mR-O complex figure copying scores (*p*< 0.05; Table 2). Since cognitive decline has been supposed to be loosely associated with the magnitude of A*β* plaque load [9], we did not use seed 18F-AV-45 SUVr to correlate with memory performances.

This exploratory analysis served to better establish the clinical impacts of entorhinal and DC seed volumes anchored by syndrome-related SN [4]. We next verified our assumption that alteration in FNs anchored to entorhinal and DC seeds would affect the changes in SN anchored to entorhinal and DC seeds in all patients with AD as compared to NCs.

### 3.3. Differences in SNs Anchored to Entorhinal and DC Seeds

#### 3.3.1. Decreased Structural Covariance in All Patients with AD Compared to the NCs

**Figure 1:**
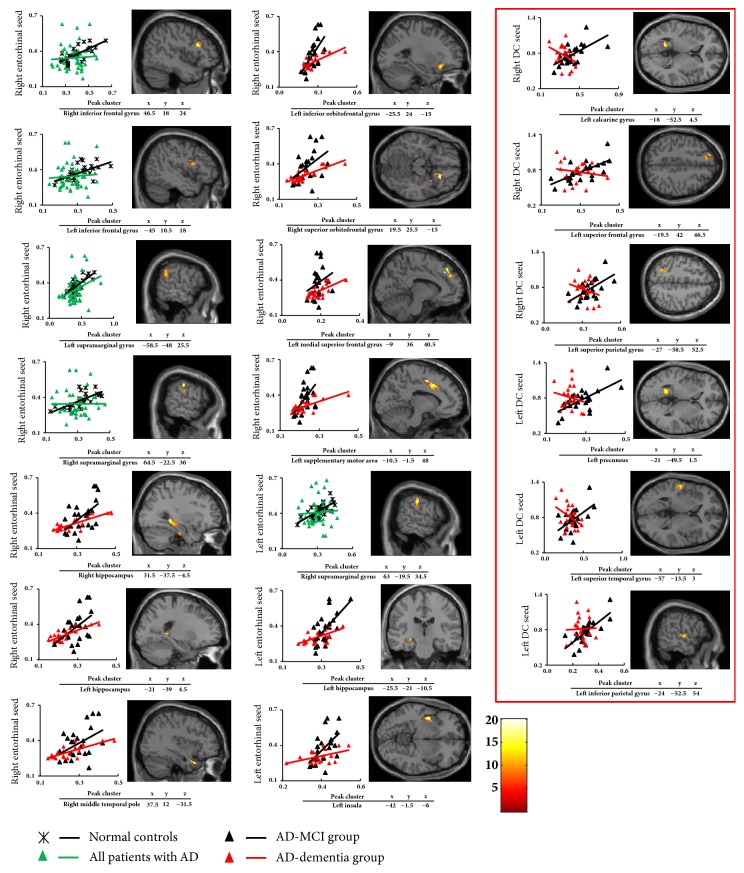
The peak clusters that showed lower structural covariance anchored to bilateral entorhinal seeds (±25, -9, -28) and bilateral DC seeds (within the red square) (±13, 15, 9) in all of the patients with AD compared to the normal controls and the AD-dementia group compared to the AD-MCI group. Correlations between GM volumes extracted from a sphere of 4 mm in radius surrounding bilateral entorhinal seeds/bilateral DC seeds and each 4 mm radius sphere around all peak voxels expressing lower structural covariance in all of the patients with AD compared to the normal controls and the AD-dementia group compared with the AD-MCI group. AD, Alzheimer's disease; DC, dorsal caudate; GM, grey matter; MCI, mild cognitive impairment.

Within the SN anchored to the right entorhinal seed, all patients with AD exhibited lower structural covariance than NCs among the regions involved in the DMN (total: 825 voxels), while within the SN anchored to the right DC seed, the comparison of structural covariance between all patients with AD and NCs did not reach the pre-established statistical threshold (Figure 1; Supplementary Table 1 in Supplementary Data).

Within the SN anchored to the left entorhinal seed, all patients with AD exhibited lower structural covariance than the NCs in the regions of the right supramarginal gyrus (total: 150 voxels), while within the SN anchored to the left DC seed, the comparison of structural covariance between all patients with AD and NCs did not reach the pre-established statistical threshold (Figure 1; Supplementary Table 2 in Supplementary Data).

#### 3.3.2. Decreased Structural Covariance in the AD-Dementia Group Compared to the AD-MCI Group

Within the SN anchored to the right entorhinal seed (Figure 1), the AD-dementia group had decreased structural covariance as compared to the AD-MCI group among the regions involved in the DMN (total: 2106 voxels; Supplementary Table 1 in Supplementary Data), while within the SN anchored to the right DC seed (Figure 1), lower structural covariance was less extended (total: 634 voxels; Supplementary Table 1 in Supplementary Data).

Within the SN anchored to the left entorhinal seed (Figure 1), the AD-dementia group had decreased structural covariance as compared to the AD-MCI group in the regions of left hippocampus and left insula (total: 879 voxels; Supplementary Table 2 in Supplementary Data), while within the SN anchored to the left DC seed (Figure 1), lower structural covariance was less extended (total: 560 voxels; Supplementary Table 2 in Supplementary Data).

### 3.4. Decreased A*β* Plaque Covariance in the AD-Dementia Group Compared to the AD-MCI Groups

**Figure 2:**
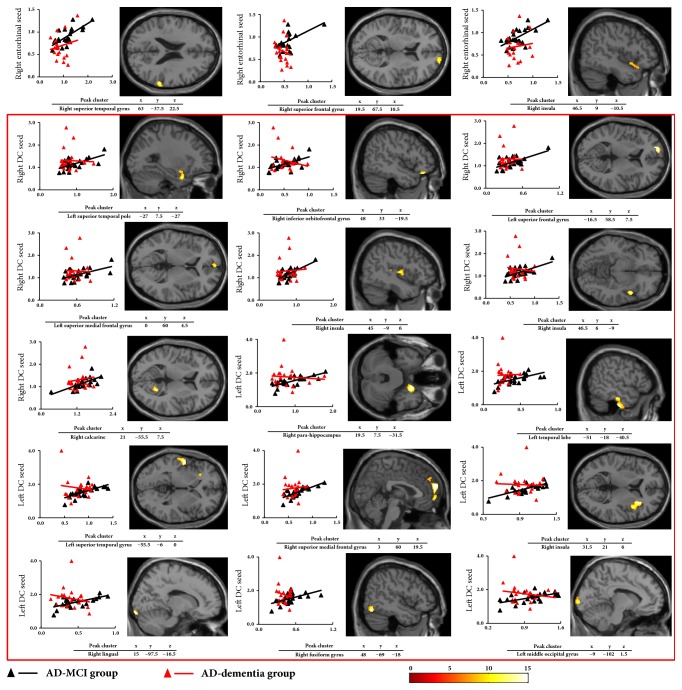
The peak clusters that showed lower A*β* plaque covariance anchored to bilateral entorhinal seeds (±25, -9, -28) and bilateral DC seeds (within the red square) (±13, 15, 9) in the AD-dementia group compared to the AD-MCI group. Correlations between 18F-AV-45 SUVr extracted from a sphere of 4 mm in radius surrounding bilateral entorhinal seeds/bilateral DC seeds and each 4 mm radius sphere around all peak voxels expressing lower A*β* plaque covariance in the AD-dementia group compared to the AD-MCI group. 18F-AV-45, florbetapir; A*β*, amyloid *β*; AD, Alzheimer's disease; DC, dorsal caudate; MCI, mild cognitive impairment; SUVr, standardized uptake value ratio.

Within AN anchored to the entorhinal seeds (Figure 2), the AD-dementia group had lower A*β* plaque covariance than the AD-MCI group in the regions of the right superior temporal and frontal gyri and right insula (total: 844 voxels; Supplementary Table 3 in Supplementary Data), while within ANs anchored to the DC seeds (Figure 2), lower A*β* plaque covariance was more extended (total: 11268 voxels; Supplementary Table 3 in Supplementary Data).

### 3.5. Difference in FN Anchored to Entorhinal and DC Seeds

#### 3.5.1. Seed-to-Voxel Analysis in All Patients with AD as Compared to the NCs

**Figure 3:**
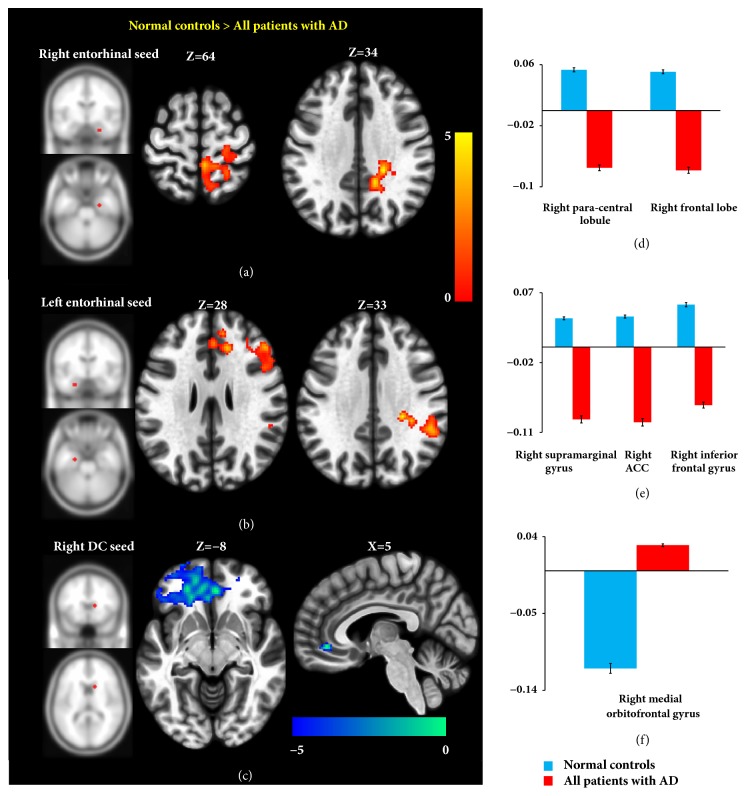
Seed-to-voxel analysis in functional networks. Left panels, maps of significant clusters showing functional connectivity with the seed regions in the right entorhinal seed (a), left entorhinal seed (b), and right DC seed (c). Red-yellow color indicates clusters demonstrating reduced functional connectivity with the seed regions in all of the patients with AD relative to the NCs; blue-white color indicates clusters demonstrating increased connectivity. Right panels, mean Fisher's transformed correlation coefficients indicating relative functional connectivity with seed regions in the right entorhinal seed (d), left entorhinal seed (e), and right dorsal caudate seed (f) for each significant cluster in all of the patients with AD and the NCs. ACC, anterior cingulate cortex; AD, Alzheimer's disease; DC, dorsal caudate; NCs, normal controls.

Within FNs anchored to entorhinal seeds, all patients with AD had significantly lower functional interregional covariance than NCs in the regions of right paracentral lobule, right frontal lobe, right supramarginal gyrus, right anterior cingulate cortex, and right inferior frontal gyrus (Figures 3(a) and 3(b)).

Within FNs anchored to DC seeds, all patients with AD did not exhibit reduced functional interregional covariance as compared to NCs. However all patients with AD had higher functional interregional covariance than NCs between the right DC seed and right medial orbitofrontal gyrus (Figure 3(c)). Figures 3(d)-3(e) show the histograms illustrating relative functional connectivity between seed regions and significant clusters.  Table 3 shows T-maxima, MNI coordinate, P value (FDR corrected), and size of each significant cluster in contiguous voxels. All patients with AD did not exhibit increased functional interregional covariance as compared to NCs within FN anchored to bilateral entorhinal and left DC seeds.

## 4. Discussion

### 4.1. Main Findings

The present study aimed to elucidate the patterns of AD biomarkers in the AD-MCI and AD-dementia groups by characterizing the changes in ANs, SNs, and FNs. There were three major findings. First, as entorhinal and DC seeds were anchored by syndrome-related ICNs [4], both seed volumes had clinical impacts. Second, all patients with AD exhibited reduced structural covariance and functional interregional covariance in networks anchored to entorhinal seeds, whereas within networks anchored to DC seeds, these patients with AD did not show any decrease in structural covariance or functional connectivity. Third, the increased connectivity within FN anchored to the right DC seed in all patients with AD as compared to NCs suggested a compensatory phenomenon within the striatal network as the disease progressed. Regarding network anchored to DC seeds, as the AD-dementia group had lower structural covariance and A*β* plaque covariance than the AD-MCI group, the decreased covariance exhibited a more extended pattern in the ANs anchored to DC seeds than in the SNs anchored to DC seeds.

### 4.2. FNs and SNs in DMN and Striatal Network

Distinct clinical syndromes have been associated with dissociable SNs [4]. As clinical features result from diverse network-based pathologic distributions, we first evaluated the clinical impact of seed volumes in the present study. AD typically follows a temporally stereotypical pattern of cognitive impairment, beginning first with episodic memory deficits, followed by dysfunction in diverse cognitive faculties including visuospatial or executive functions [34–36]. Regarding episodic memory function, the entorhinal cortex affects memory encoding, consolidation, and retrieval via its connectivity with the hippocampus and various association cortical regions [37], whereas memory under a cued response depends mostly on the DC [38]. Consistent with these observations, the right DC seed volume in the present study was shown to be specifically associated with memory under a cued response, and the entorhinal seed volume was shown to be involved with various episodic memory functions. We also observed that the DC seed volumes were associated with more widespread cognitive functions including visuospatial and executive performance. DC atrophy and its decreased connectivity with frontal cortices have been linked to executive dysfunction [39]. Although no previous studies have reported an association between DC volume and visuospatial function, lower DC dopamine levels have been associated with greater dysfunction in visuospatial skills [40]. As functional connectivity in striatal networks anchored to DC seeds has been shown to be lower in later disease stages [11], impairment of the DC seed volume-related cognitive function in our data may have occurred in later stages of AD as a result of striatal network disconnection. This is generally consistent with the literature showing that dysfunction in cognitive processes other than memory temporally follows memory deficits [34–36]. The clinical-pathological relationships of each seed volume suggest that network degeneration also had a clinical impact.

Regarding SNs and FNs, all patients with AD exhibited reduced structural covariance and functional interregional covariance between right entorhinal seed and the regions of frontal lobes and between left entorhinal seed and the regions of supramarginal gyri as compared to NCs. In addition, all patients with AD, relative to NCs, did not demonstrate reduced structural covariance or functional interregional covariance within the network anchored to DC seeds. Corresponding patterns, rather than completely overlap, between altered SN and FN were detected. Some discrepancies may be attributed to the complex mechanisms underlying anatomical and functional interregional covariance, such as developmental, genetic, and environmental factors [41].

The pathologic trajectory of AD suggests that structural topological disorganization of the brain occurs after the stages of accelerated NFT deposition [9]. The cascading network process in AD proposes that functional connectivity overload is followed by functional failure in DMN during the preclinical stage of AD [14]. The acceleration of NFT accumulation has been attributed to high basal metabolism in the interconnected regions [4] involved in the overloaded DMN [14]. NFT deposition along the DMN has been associated with FN overload in preclinical stage of AD [14]; however how alterations in FNs affect anatomic atrophy remains elusive. Regarding temporal evolution, acceleration of NFT accumulation precedes the acceleration of atrophy rates in some brain regions [9]. Such atrophy has been shown to be associated with decreased structural covariance among the hubs of DMN in early clinical stage of AD [6]. Our observations are generally consistent with the literatures [6, 9] and expand some of the previous findings. Our study indicates that the decreased structural covariance in DMN accelerates upon functional failure in the DMN and that network failure in both SN and FN occurs after the FN overload-related NFTs deposition. The direct corresponding patterns between the reduction in structural covariance and functional failure within DMN suggest a FN failure-associated SN disruption in early clinical stage of AD.

Within the patients with early clinical stage of AD presenting with A*β* plaques, decreased structural covariance in SNs anchored to DC seeds exhibited restricted patterns as the disease progressed. Meanwhile, these AD patients had a compensatory increase in functional connectivity in FN anchored to right DC seed as compared to NCs. With regard to the findings of high interregional covariance within FNs anchored to DC seeds, such increased metabolic demands may be associated with accelerated NFT propagation along the hubs involved in the striatal network [4]. A more pronounced alteration in SN anchored to DC seeds may be prominent in the later clinical stage of AD.

Functional failure in the DMN was associated with an extended pattern of reduced structural covariance in SN anchored to entorhinal seeds, while the increased connectivity in FNs anchored to DC seeds was associated with a restricted pattern of decreased structural covariance within SN anchored to DC. The patterns of decreased structural covariance in AD patients as the disease progresses are generally tracking the network alteration in ICNs as revealed by fMRI.

### 4.3. FNs and ANs in the DMN and Striatal Network

The deposition of A*β* plaque has been associated with high excitatory synaptic activity in the interconnected regions of ICNs [3]. It has been suggested that this high basal metabolism triggers downstream A*β* plaque deposition events [42]. Consistent with previous studies, as all of our patients with AD had a compensatory increased connectivity within FNs anchored to DC seeds, the AD-dementia group exhibited an extended pattern of reduced A*β* plaque covariance in ANs anchored to DC seeds. We also demonstrated a steeper slope of accelerated A*β* plaque accumulation within the striatal network as the disease progressed in the stage of mild AD-dementia.

In contrast to the findings regarding the striatal network, the pattern in the DMN revealed a plateau phenomenon of A*β* plaque deposition within AN anchored to entorhinal seeds. Other studies have shown that A*β* plaque accumulation along the regions involved in the DMN is only apparent in the preclinical stage of AD [3, 43]. The curve representing A*β* plaque deposition has been reported to show a sigmoid-curved shape and to reach a plateau in the early clinical stage of AD [9, 44, 45]. The observation that the AD-dementia group had a restricted pattern of reduced A*β* plaque covariance in AN anchored to entorhinal seed is generally consistent with the theoretic model of pathologic change in AD [9]. We further demonstrated that such decelerated A*β* plaque accumulation along the hubs involved in DMN became apparent on DMN disconnection in the clinical stage of mild AD-dementia.

Functional failure in the DMN was associated with a restricted pattern of reduced A*β* plaque covariance in ANs anchored to entorhinal seeds, while the increased connectivity in FNs anchored to DC seeds was associated with an extensive pattern of decreased A*β* plaque covariance within ANs anchored to DC seeds. ICNs-associated spatial distribution should be considered when demonstrating temporal evolution of the pathologic changes [9].

### 4.4. FNs within DMN and Striatal Network

Mechanisms underlying the changes in FNs anchored to DC seeds in AD are less well-established than those underlying the DMN. However, studies on Parkinson's disease (PD) have emphasized the critical role of the striatal network in cognitive impairment [46]. Decreased functional connectivity between DC seeds and regions including the lateral prefrontal gyrus and posterior parietal cortex has been correlated with executive dysfunction [47, 48]. Pathologically, the PD dementia-associated functional failure in the striatal network has been attributed to A*β* plaque deposition in the DC [49]. As A*β* plaque deposition in the striatum has been observed in the later clinical stage of AD [50], functional failure in striatal network has been postulated to be less pronounced in the early clinical stage.

As functional connectivity declines in the DMN [5], increasing connectivity in other ICNs plays a compensatory role until reaching a functional overload, which subsequently leads to a network-based functional decline in the overloaded ICNs [10]. In agreement with this observation, all of our patients with AD had failure in FNs anchored to entorhinal seed as compared to the NCs, with simultaneous increased functional connectivity in FNs anchored to DC seeds. Across the entire AD spectrum, the shifting in systems-level ICNs has been referred to as cascading network failure [14, 15]. Striatal network could be one of the ICNs which plays a compensatory role when DMN undergoes functional failure [42].

## 5. Limitations

This study has several limitations. First, there are limitations inherent to its cross-sectional design, and follow-up studies investigating how different large-scale ICNs affect pathologic evolution across a broader AD spectrum are needed. Second, we only characterized the pathologic and functional patterns of the DMN anchored to entorhinal seeds. Future studies investigating the progression of structural covariance and A*β* plaque covariance in subsystems of the DMN and striatal networks may help clarify the role of each FN and network-based spatial pathologic changes. Third, the clinical use with 18F-AV-45 PET with regard to networks of A*β* plaques requires further pathologic investigations. However, the regional hierarchy of amyloid deposition signals shown in 18F-AV-45 PET suggests its role in* in vivo* staging of the progression of amyloid pathology [51].

## 6. Conclusions

We propose that alterations in FNs may affect changes in SNs and ANs. Our model suggested that the DMN fails during the development of AD-dementia and that processing burden is then shifted to striatal network. The accelerated molecular pathologic changes in the regions involved in the ICNs were attributed to increased functional connectivity in the corresponding ICNs. The compensatory increased functional connectivity within the striatal network was associated with acceleration in the decreased A*β* plaque covariance within ANs anchored to DC seeds. Accelerated deposition of NFTs attributed to high functional connectivity in the DMN was followed by atrophy, and SN anchored to entorhinal seeds exhibited acceleration in the reduced structural covariance as the DMN underwent functional failure. The temporal trajectory of pathologic change can be well demonstrated in pathologic covariance among different regions involved in ICNs. Pathologic covariance provides critical support to the pathologic interactions at network and molecular level.

## Figures and Tables

**Table 1:** Demographic data of the study participants.

	Normal controls	AD-MCI	AD-dementia
Male/Female	17/13	12/11	11/9

Age (years)	65.9±5.6	71.4±8.1	70.0±6.7

Education (years)	9.0±4.8	10.8±4.1	7.7±5.4 ^b^

**Cognitive test score**			

Mini-mental state examination	27.1±3.2	26.3±2.3	19.0±4.7 ^ab^

Episodic memory scores			

CVVLT-10 min	7.2±1.5	5.87±2.4 ^a^	1.5±2.1 ^ab^
CVVLT-cued	7.3±1.3	6.2±2.2 ^a^	1.9±2.1 ^ab^

Executive function scores			

Trail Making Test parts B	11.7±4.3	11.7±3.2	5.3±4.8 ^ab^
Stroop interference	35.3±12.4	28.7±15.7	18.1±12.7 ^ab^

Visuospatial function scores			

VOSP	8.1±2.4	8.3±2.0	4.6±3.5 ^ab^
mR-O complex figure copying	16.3±2.2	16.7±0.9	13.8±6.0 ^ab^

**TIV adjusted VOI volume ** **∗** **10** ^**-3**^			

Right hippocampus	1.5±0.1	1.4±0.2 ^a^	1.1±0.2 ^ab^
Left hippocampus	1.4±0.1	1.4±0.2 ^a^	1.1±0.2 ^ab^

Values with mean±standard deviation. ^a^*p*< 0.05, significant difference compared to normal controls using the independent *t*-test. ^b^*p*< 0.05, significant difference compared to AD-MCI using the independent *t*-test in AD-dementia. AD, Alzheimer's disease; CVVLT-10 min, Chinese version of the Verbal Learning Test with 10 minutes' interval recall; CVVLT-cued, Chinese version of the Verbal Learning Test-cued recall; MCI, mild cognitive impairment; mR-O, modified Rey–Osterrieth; TIV, total intracranial volume; VOI, volume of interest; VOSP, visual object and space perception.

**Table 2:** Correlation between scores of memory performance tests and seed volumes.

Seed	Right entorhinal	Left entorhinal	Right DC	Left DC
Dependent variable	**ρ** ** value**	**ρ** ** value**	**ρ** ** value**	**ρ** ** value**
	**(*p* value)**	**(*p* value)**	**(*p* value)**	**(*p* value)**

Episodic memory scores				
CVVLT-10min	**0.356**	0.119	0.186	0.110
	**(0.002)**	(0.319)	(0.118)	(0.385)
CVVLT-cued	**0.301**	0.117	**0.285**	0.185
	**(0.010)**	(0.326)	**(0.015)**	(0.119)

Executive function scores				
Trail Making Test parts B	0.129	0.096	**0.239**	**0.233**
	(0.280)	(0.421)	**(0.043)**	**(0.049)**
Stroop interference	**0.302**	0.215	**0.253**	**0.363**
	**(0.010)**	(0.069)	**(0.032)**	**(0.002)**

Visuospatial function scores				
VOSP	0.074	-0.07	0.126	0.070
	(0.539)	(0.560)	(0.291)	(0.560)
mR-O complex figure copying	0.141	-0.020	**0.314**	**0.283**
	(0.236)	(0.867)	**(0.007)**	**(0.016)**

Partial correlations controlled for diagnosis. Values with *ρ* value (*p* value). Bold font indicates statistical significance at *p*< 0.05. CVVLT-10 min, Chinese version of the Verbal Learning Test with 10 minutes' interval recall; CVVLT-cued, Chinese version of the Verbal Learning Test-cued recall; DC, dorsal caudate; mR-O, modified Rey–Osterrieth; VOSP, visual object and space perception.

**Table 3:** Seed-to-voxel analysis, brain regions showing different alterations to functional connectivity between that anchored to entorhinal seeds and that anchored to DC in all of the patients with AD compared to NCs.

	**Seed**	**Contrast**	**Cluster**	**MNI (x, y, z)**	**Cluster size**	**T**	**p-FDR**
Entorhinal seed	Right	NCs> all of the patients with AD	Right paracentral lobule	4, -40, 64	677	4.46	<0.0001
			Right frontal lobe	22, -34, 34	342	5.86	<0.0001
		All of the patients with AD > NCs	No peak cluster				
	Left	NCs> all of the patients with AD	Right supramarginal gyrus	52, -40, 32	609	4.82	0.000024
			Right anterior cingulate cortex	14, 32, 28	225	4.19	0.000078
			Right inferior frontal gyrus	54, 20, 30	200	4.21	0.000078
		All of the patients with AD > NCs	No peak cluster				

DC seed	Right	NCs> all of the patients with AD	No peak cluster				
		All of the patients with AD > NCs	Right medial orbitofrontal gyrus	2, 42, -8	1173	5.02	0.000004
	Left	NCs> all of the patients with AD	No peak cluster				
		All of the patients with AD > NCs	No peak cluster				

MNI coordinates (x, y, z), T maxima, and contiguous voxels of cluster size are shown. The significance clusters are detected with a threshold of FDR corrected *p*< 0.05 at cluster level and uncorrected* p*< 0.001 at voxel level. AD, Alzheimer's disease; DC, dorsal caudate; FDR, false discovery rate; MCI, mild cognitive impairment; MNI, Montreal Neurological Institute; NCs, normal controls.

## Data Availability

The datasets used and/or analyzed during the current study are available from the corresponding author on reasonable request.

## Supplementary Materials

Supplementary Materials Supplementary Table 1: group difference in structural network anchored to the right entorhinal and right DC seeds. Supplementary Table 2: group difference in structural network anchored to the left entorhinal and left DC seeds. Supplementary Table 3: decreased *β*-amyloid plaque covariance in AD-dementia compared with AD-MCI in amyloid network anchored to the bilateral entorhinal and DC seeds.
