# Supplementary material for: Altered Functional Network Affects Amyloid and Structural Covariance in Alzheimer's Disease
Source: Biomed Res Int. 2018 Dec 2;2018:8565620. doi: 10.1155/2018/8565620 (PMC6304529; doi:10.1155/2018/8565620)
Supplement: Supplementary Materials — Supplementary Table 1: group difference in structural network anchored to the right entorhinal and right DC seeds. Supplementary Table 2: group difference in structural network anchored to the left entorhinal and left DC seeds. Supplementary Table 3: decreased β-amyloid plaque covariance in AD-dementia compared with AD-MCI in amyloid network anchored to the bilateral entorhinal and DC seeds. [file 8565620.f1.docx]

**Supplementary Table 1** Group difference in structural network anchored to the right entorhinal and right DC seeds

| **Right entorhinal Seed: 25,-9,-28** | x | y | z | Cluster size (voxels) | F-score |
| --- | --- | --- | --- | --- | --- |
| Decreased structural covariance in all AD compared with NCs | | | | | |
| Right inferior frontal gyrus | 46.5 | 18 | 24 | 291 | 18.7486 |
| Left inferior frontal gyrus | -45 | 10.5 | 18 | 165 | 12.6652 |
| Left supramarginal gyrus | -58.5 | -48 | 25.5 | 220 | 13.9258 |
| Right supramarginal gyrus | 64.5 | -22.5 | 36 | 149 | 15.2087 |
| Decreased structural covariance in AD-dementia compared with AD-MCI | | | | | |
| Right hippocampus | 31.5 | -37.5 | -4.5 | 702 | 25.7689 |
| Left hippocampus | -21 | -39 | 4.5 | 120 | 13.6499 |
| Right middle temporal pole | 37.5 | 12 | -31.5 | 359 | 13.5474 |
| Left inferior orbitofrontal gyrus | -25.5 | 24 | -15 | 135 | 15.4474 |
| Right superior orbitofrontal gyrus | 19.5 | 25.5 | -15 | 441 | 24.1932 |
| Left medial superior frontal gyrus | -9 | 36 | 40.5 | 123 | 19.7836 |
| Left supplementary motor area | -10.5 | -1.5 | 48 | 226 | 28.5458 |
| **Right DC Seed: 13,15,9** |  |  |  |  |  |
| Decreased structural covariance in all AD compared with NCs | | | | | |
| No peak clusters |  |  |  |  |  |
| Decreased structural covariance in AD-dementia compared with AD-MCI | | | | | |
| Left calcarine | -18 | -52.5 | 4.5 | 295 | 16.4526 |
| Left superior frontal gyrus | -19.5 | 42 | 46.5 | 201 | 13.4422 |
| Left superior parietal gyrus | -27 | -58.5 | 52.5 | 138 | 14.5881 |

Significance with a threshold of *p*< 0.01 at cluster level and *p*< 0.05 for family-wise error correction at voxel level and cluster size> 100 voxels. AD, Alzheimer’s disease; DC, dorsal caudate; MCI, mild cognitive impairment; NCs, normal controls.

**Supplementary Table 2** Group difference in structural network anchored to the left entorhinal and left DC seeds

| **Left entorhinal Seed: -25,-9,-28** | x | y | z | Cluster size (voxels) | F-score |
| --- | --- | --- | --- | --- | --- |
| Decreased structural covariance in all AD compared with NCs | | | | | |
| Right supramarginal gyrus | 63 | -19.5 | 34.5 | 150 | 16.2251 |
| Decreased structural covariance in AD-dementia compared with AD-MCI | | | | | |
| Left hippocampus | -25.5 | -21 | -10.5 | 106 | 12.9743 |
| Left insula | -42 | -1.5 | -6 | 773 | 25.7232 |
| **Left DC Seed: -13,15,9** |  |  |  |  |  |
| Decreased structural covariance in all AD compared with NCs | | | | | |
| No peak cluster |  |  |  |  |  |
| Decreased structural covariance in AD-dementia compared with AD-MCI | | | | | |
| Left precuneus | -21 | -49.5 | 1.5 | 253 | 15.3305 |
| Left superior temporal gyrus | -57 | -13.5 | 3 | 190 | 13.0839 |
| Left inferior parietal gyrus | -24 | -52.5 | 54 | 117 | 14.2519 |

Significance with a threshold of *p*< 0.01 at cluster level and *p*< 0.05 for family-wise error correction at voxel level and cluster size >100 voxels. AD, Alzheimer’s disease; DC, dorsal caudate; MCI, mild cognitive impairment; NCs, normal controls.

**Supplementary Table 3** Decreased β-amyloid plaque covariance in AD-dementia compared with AD-MCI in amyloid network anchored to the bilateral entorhinal and DC seeds

| **Right entorhinal Seed: 25,-9,-28** | x | y | z | Cluster size (voxels) | F-score |
| --- | --- | --- | --- | --- | --- |
| Right superior temporal gyrus | 63 | -37.5 | 22.5 | 299 | 13.5201 |
| Right superior frontal gyrus | 19.5 | 67.5 | 10.5 | 352 | 11.4713 |
| Right insula | 46.5 | 9 | -10.5 | 193 | 9.2951 |
| **Left entorhinal Seed: -25,-9,-28** | | | | | |
| No peak cluster |  |  |  |  |  |
| **Right DC: 13,15,9** |  |  |  |  |  |
| Left superior temporal pole | -27 | 7.5 | -27 | 437 | 12.4746 |
| Right inferior orbitofrontal gyrus | 48 | 33 | -19.5 | 108 | 11.5728 |
| Left superior frontal gyrus | -16.5 | 58.5 | 7.5 | 178 | 19.047 |
| Left superior medial frontal gyrus | 0 | 60 | 4.5 | 130 | 10.5777 |
| Right insula | 45 | -9 | 6 | 287 | 11.878 |
| Right insula | 46.5 | 6 | -9 | 120 | 13.8988 |
| Right calcarine | 21 | -55.5 | 7.5 | 140 | 13.4964 |
| **Left DC: -13,15,9** |  |  |  |  |  |
| Right para-hippocampus | 19.5 | 7.5 | -31.5 | 2641 | 15.4959 |
| Left temporal lobe | -51 | -18 | -40.5 | 473 | 15.8612 |
| Left superior temporal gyrus | -55.5 | -6 | 0 | 3416 | 17.0668 |
| Right superior medial frontal gyrus | 3 | 60 | 19.5 | 2011 | 22.2073 |
| Right insular | 31.5 | 21 | 6 | 682 | 17.7961 |
| Right lingual gyrus | 15 | -97.5 | -16.5 | 110 | 17.2521 |
| Right fusiform gyrus | 48 | -69 | -18 | 319 | 15.3593 |
| Left middle occipital gyrus | -9 | -102 | 1.5 | 216 | 12.4658 |

Significance with a threshold of *p*< 0.01 at cluster level and *p*< 0.05 for family-wise error correction at voxel level and cluster size >100 voxels. AD, Alzheimer’s disease; DC, dorsal caudate; MCI, mild cognitive impairment.
